# Genome-wide CRISPR/Cas9-knockout in human induced Pluripotent Stem Cell (iPSC)-derived macrophages

**DOI:** 10.1038/s41598-021-82137-z

**Published:** 2021-02-19

**Authors:** Elena Navarro-Guerrero, Chwen Tay, Justin P. Whalley, Sally A. Cowley, Ben Davies, Julian C. Knight, Daniel Ebner

**Affiliations:** 1 Nuffield Department of Medicine, Target Discovery Institute, University of Oxford, Oxford, UK; 2 Wellcome Centre for Human Genetics, University of Oxford, Oxford, UK; 3 James Martin Stem Cell Facility, Sir William Dunn School of Pathology, University of Oxford, Oxford, UK

**Keywords:** Induced pluripotent stem cells, Functional genomics, Target identification

## Abstract

Genome engineering using CRISPR/Cas9 technology enables simple, efficient and precise genomic modifications in human cells. Conventional immortalized cell lines can be easily edited or screened using genome-wide libraries with lentiviral transduction. However, cell types derived from the differentiation of induced Pluripotent Stem Cells (iPSC), which often represent more relevant, patient-derived models for human pathology, are much more difficult to engineer as CRISPR/Cas9 delivery to these differentiated cells can be inefficient and toxic. Here, we present an efficient, lentiviral transduction protocol for delivery of CRISPR/Cas9 to macrophages derived from human iPSC with efficiencies close to 100%. We demonstrate CRISPR/Cas9 knockouts for three nonessential proof-of-concept genes—*HPRT1, PPIB* and *CDK4*. We then scale the protocol and validate for a genome-wide pooled CRISPR/Cas9 loss-of-function screen. This methodology enables, for the first time, systematic exploration of macrophage involvement in immune responses, chronic inflammation, neurodegenerative diseases and cancer progression, using efficient genome editing techniques.

## Introduction

CRISPR/Cas9 genome-wide screening represents a huge step forward in our ability to systematically probe human cellular physiology on a genome-wide scale. High-throughput screening techniques using CRISPR/Cas9 pooled libraries targeting the entire human genome enable research scientists to probe gene function in an un-biased manner and to investigate an extensive range of biological processes. Indeed, since the relatively recent introduction of such CRISPR/Cas9 libraries from the Zhang^1^, Weissman^2^, and Moffat laboratories^3^ among others, these libraries have been used in numerous screening applications, outperforming previous genome-wide screening tools such as RNAi^4^. Much of the utility of these libraries stems from the use of lentiviral transduction protocols to precisely and efficiently introduce on average one CRISPR/Cas9 vector per cell^4,5^ and this has been achieved for many experimental human cell types including immortalized cell lines^1,3,6–8^ and patient derived cells^9,10^. Parallel to these improvements in precision genome engineering has been the development of technologies to generate human induced Pluripotent Stem Cells (iPSC) by reprogramming somatic cells (usually fibroblasts) that have been harvested from patients or healthy donors^11^. iPSC have revolutionised our ability to model human genetic disease over the past decade as they retain the donor’s genotype, can replicate indefinitely, can be produced at scale and can be instructed to differentiate into a broad variety of cell types. These models are then employed to interrogate and experimentally perturb human physiology. They are particularly powerful tools where the analogous tissue or cell type from human patients is inaccessible or in limited supply. Importantly, iPSC are the only model that can recapitulate the complex genetics of polygenic disease where multiple mutations in critical pathways tip the balance of risk towards developing disease. In contrast, immortalized cell lines frequently have atypical cell cycles and highly abnormal karyotypes, factors which complicate studies where correct gene dosage and molecular stoichiometry are critical. To date, most CRISPR/Cas9 genome-wide screens have been conducted in immortalized cell lines, but there is considerable interest in developing efficient, cost-effective protocols for performing screens in more biologically relevant iPSC models. Undifferentiated iPSC genome-wide CRISPR/Cas9 screens followed by differentiation have been developed^12,13^ and there have been recent examples of genome-wide screens in differentiated iPSC^14^, but to our knowledge, no genome-wide screening method to systematically probe differentiated macrophages from iPSC has been reported.

Macrophages and specialized tissue-specific macrophages, such as microglia, are innate immune cells that play a crucial role in the response to pathogens, and also have homeostatic roles in tissues, phagocytosing and digesting microbes and foreign material, proteinaceous and cellular debris, incompetent synapses, apoptotic and cancerous cells^15–17^. We have previously developed methods for generating iPSC-derived macrophages of high purity and functionality^18,19^ and others have developed similar protocols (reviewed by Lee^20^). These macrophages differentiate independently of *MYB* expression (required for definitive haematopoiesis), therefore represent primitive, tissue-type macrophages^21^. They have been used to model HIV infection^18^, genetic immunodeficiency^22^, phagocytosis^23^, and can be further differentiated to microglia to model neuroinflammation in Alzheimer’s disease^23^. However, the difficulty of delivery methods for efficient CRISPR/Cas9 editing of iPSC-derived macrophages^24^ has hindered the application of genome-wide CRISPR screens in these cells. CRISPR/Cas9 screens have been performed in the immortalized THP1 cell line^25^ which, although useful in many research applications, are considered a poor model of mature primary macrophages^26^. Here we present a new method of achieving efficient lentiviral transduction of fully functional iPSC-derived macrophages, allowing, for the first time, CRISPR/Cas9 gene editing tools to be used cost-effectively within this cell type. This method can be easily scaled and applied to drug screening or to develop genome-wide and targeted screening approaches with great therapeutic potential.

## Results

### Characterisation of iPSC-derived macrophages

We differentiated macrophages from a published control iPSC line SFC841-03-01^27^ and SFC180-01-01^23^ following our previously published protocol^18^ (Fig. S1A—iPSC precursors and iPSC-differentiated macrophages). We first sought to confirm the macrophage phenotype by flow cytometry using conjugated antibodies for CD11b (integrin alpha M, a marker for mature myeloid cells and a subunit of the complement receptor, CR3 or Mac-1), CD14 (a component of the receptor for bacterial lipopolysaccharide (LPS)), CD80 (membrane receptor of CD28 or CTLA-4, a specific marker of human M1 macrophages), and CD163 (an acute phase-regulated receptor, a marker of monocytes and macrophages). This demonstrated that the differentiated cultures produced a homogenous population of iPSC-derived macrophages CD11b^+^ CD14^+^ CD80^low^ CD163^low^ (Fig. S1B). To confirm the innate immune activation capacity of the differentiated macrophages, cells were then stimulated with LPS and the pro-inflammatory cytokine TNFα measured by intracellular cytokine staining using flow cytometry. We found that the average level of TNF positive cells was 75.1 ± 6.5% (versus 0.1 ± 0.3% for unstimulated cells), demonstrating these cells have a robust induced TNF response (Fig. S1C).

### Efficient gene knockout in iPSC-derived macrophages

To assess the efficiency of Cas9-editing in iPSC-derived macrophages, we targeted three macrophage non-essential genes; hypoxanthine–guanine phosphoribosyl-transferase 1 (*HPRT1*), peptidylprolyl isomerase B (*PPIB*) and cyclin dependent kinase 4 (*CDK4*), expression of which has been confirmed by transcriptome analysis^16^. Each of these genes was targeted specifically with a single sgRNA or a pool of four sgRNAs from the TKOv3 library^3^. We chose this library because of an improved guide RNA design^28^ over similar libraries and it has been previously validated in genome-wide loss of function-screens^7,9,28,29^. Moreover, the TKOv3 library targets 18,053 protein-coding genes with four sgRNAs per gene, which is fewer guides than other systems^1,30^ and it is particularly useful for running genome-wide CRISPR screens where cell numbers are limiting due to expense, as with iPSC macrophage models. We aimed to deliver the sgRNA and Cas9 by lentiviral transduction using the lentiCRISPRv2 backbone^1^ which is a single-vector system with an expression cassette for a single gRNA and Cas9 on the same vector, and includes a puromycin resistance gene to allow purification of the transduced cells by antibiotic selection. Since human macrophages restrict lentiviral infection^31^, we reasoned that co-incubation of the sgRNA/Cas9 lentivirus with Vpx virus-like particles (Vpx-VLPs)^32^ together with each CRISPR lentivirus would deliver increased efficiency as Vpx causes degradation of the macrophage lentiviral restriction factor, SAMHD1^33^, thereby facilitating transduction and enabling the genetic modification of macrophages^34^. We tested SAMHD1 degradation with increasing amounts of Vpx-VLPs in Vpx-transduced macrophages, and we confirmed that Vpx-VLPs depleted SAMHD1 protein in iPSC-derived macrophages (Fig. S2A). We carried out transductions in the presence of polybrene since this reagent has been shown to facilitate viral attachment to the cell surface^35^ and has been used successfully in CRISPR/Cas9 genome-wide screening^3,10,29^. Finally, we tested plate-infection versus spinfection (alternatively called spinoculation). We found the method that showed highest efficiency was spinfection with polybrene and Vpx (Fig. S2B). We optimised puromycin selection of transduced cells, as antibiotic selection is routinely employed in lentiviral transduction protocols to obtain a pure transduced population. We determined the optimal concentration by a tolerance test of each macrophage harvest, using puromycin-supplemented media for 10 days, with media changes every 3–4 days (Fig. S2C). We found the majority of non-transduced macrophages died when exposed to 1 µg/mL puromycin. Whilst optimizing the puromycin concentration, we observed that puromycin-treated transduced macrophages exhibited a decrease in TNF expression upon LPS stimulation from 71.9 ± 8.0 to 42.2 ± 7.0% when quantified by flow cytometry (Fig. S2E). One explanation for the lower levels of TNF^+^ cells is that the transduced cells (puromycin-resistant) may have a suppressed production of pro-inflammatory mediators, most likely due to ingestion of dead cells, which is thought to be anti-inflammatory and immunosuppressive^15,36,37^.

**Figure 1:**
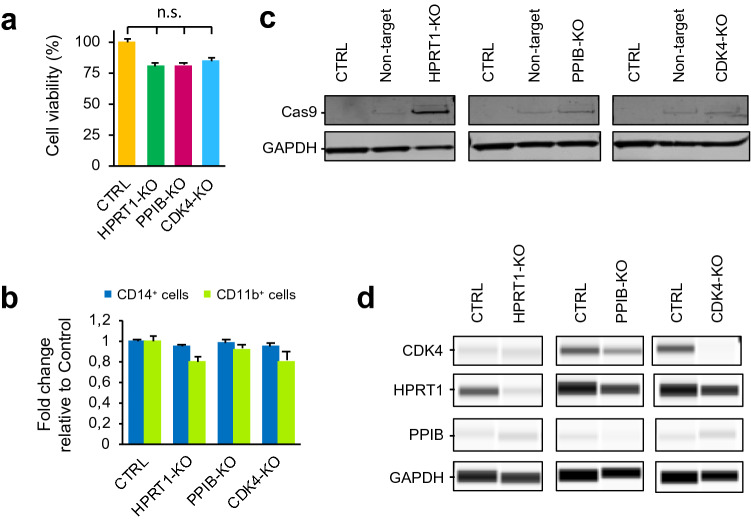
iPSC-derived macrophage knock-out targeting three macrophage non-essential genes: HPRT1; PPIB and CDK4. (**a**) Percentage of cell viability (±SEM) in iPSC-derived macrophages 3 days after transduction with pool of 4 sgRNAs of HPRT1, PPIB and CDK4 lentiviruses. CTRL sample are cells after spinfection with PB and Vpx. (**b**) Fold change (±SEM) of CD14+ and CD11b+ iPSC-derived macrophages 14 days after transduction with HPRT1, PPIB and CDK4 lentiviruses. (**c**) Western blot analysis of Cas9 protein in CRISPR/Cas9-transduced iPSC-derived macrophages. GAPDH loading control. (**d**) Detection of HPRT1, PPIB and CDK4 in capillary western blot (Wes) assay in transduced iPSC-derived macrophages (MOI 3). GAPDH loading control. Full length gels and blots are included in Supplementary Figs. S5 and S6. Capillary Wes images were obtained from Compass for SW 5.0.1. software. https://www.proteinsimple.com/compass. *n.s.* non-significant difference.

We then proceeded to perform macrophage transductions at multiplicity of infection (MOI) 1 for individual guides or MOI 3 for pooled guides. We transduced differentiated macrophages from 6 to 8 weeks-old differentiation cultures, then allowed the cells to complete macrophage maturation for 14 days using M-CSF (Macrophage Colony-Stimulating Factor) supplemented media. We monitored cell survival at day 3 post-transduction, and no significant effect on cell viability was observed after any of the three gene transductions (Fig. 1A). The transduced cells were then selected with puromycin for 11 days. We validated the differentiation phenotype by flow cytometry 14 days after transduction and found the percentage of CD14^+^ or CD11b^+^ cells did not change substantively after transduction with the pooled guides (Figs. 1B, S3), confirming that differentiation of iPSC-derived macrophages was not affected by the transduction.

Next, we quantified the knockout efficiency of the transduction by western blotting. We found the pool of four sgRNAs achieved a strong knockdown of each of the three genes tested (Figs. 1C,D, S5, S6). We validated the editing of a single sgRNA at MOI 1 using the recently developed TIDE (Tracking of Indels by Decomposition)^38^ protocol to analyse the editing efficiency of individual sgRNAs. The cutting efficiencies of the guides varied from 2 to 80%, similar to the range observed in previous methods^39^, and all guides produced indels at the expected break sites (Fig. S2F).

### Delivery of genome-wide knockout library into iPSC-derived macrophages

**Figure 2:**
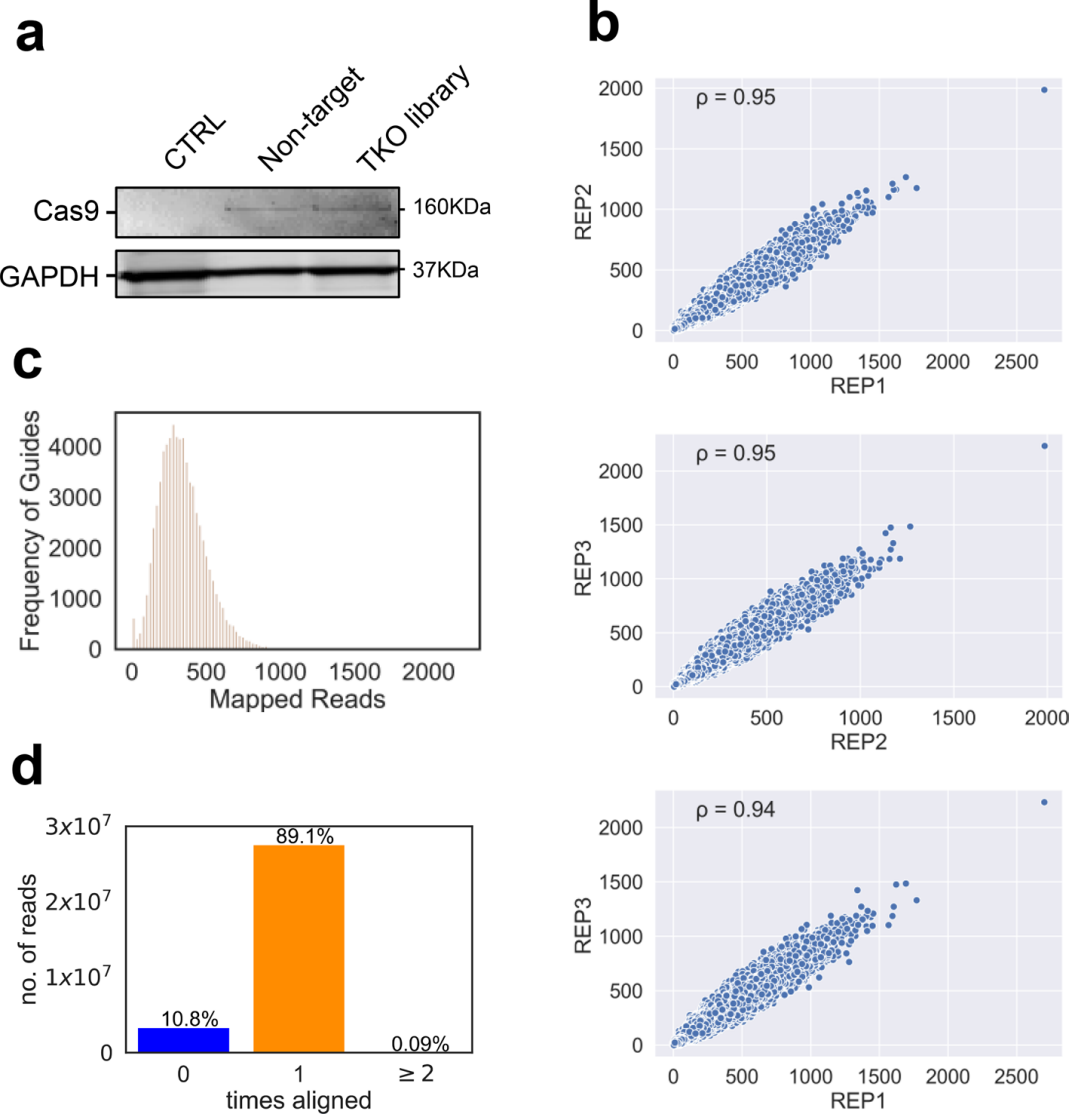
Delivery of genome-wide knockout library to iPSC-derived macrophages. (**a**) Western blot analysis of Cas9 expression in TKOv3-transduced cells using anti-Cas9 antibody detected an ~ 160 kDa band. GAPDH loading control. (**b**) Scatter plots comparing the abundance of each of the guides sequenced for each of the replicates. Pearson's correlation coefficient is reported for each pairwise comparison, with the correlation coefficient > 0.9 for each comparison. (**c**) Histogram showing the distribution of reads for the guides, with a mean of 339 reads per guide and a maximum of 2233 reads per guide. (**d**) Graph showing mapped sequences of the gDNA to the TKOv3 library. We found 89.1% of the sequences mapped to one guide in the library, 10.8% of the sequences were mapped to nothing in the library and 0.09% of the guides mapped to two or more guides in the library.

After obtaining confirmation of efficient single gene lentiCRISPR knockout in iPSC derived macrophages, we proceeded to perform a proof of principle genome-wide transduction of a CRISPR/Cas9 library to validate the delivery of a genome-wide CRISPR/Cas9 library to iPSC derived macrophages (Fig. S2D). CRISPR/Cas9 genome-wide screens in iPSC-derived macrophages would be a valuable tool to interrogate the biology of these innate immune cells so to test this, we transduced iPSC-derived macrophage precursors with the TKOv3 CRISPR lentiviral library (pool of 71,090 guides) at MOI 1 and to establish statistical power for delivery evaluation, a coverage of 250 cells per guide was maintained throughout the experiment. The cells were then incubated for 14 days to complete maturation. To retain the maximally responsive cellular phenotype as a potential screening platform, we decided not to subject the TKO3-transduced macrophages to puromycin to avoid the decrease in TNF expression upon LPS stimulation of the macrophages following the antibiotic selection process that we observed during single-guide sequence transductions and selection. We assessed Cas9 expression in the TKOv3-transduced macrophages by western blotting (Figs. 2A, S4). We conducted three biological replicate transductions of the TKOv3 library, collected 40.0E6 cells from each and then extracted genomic DNA, PCR-amplified the inserted guides and performed Next-Generation sequencing on a HiSeq4000. This revealed an excellent replicate correlation of ≥ 0.94 for each comparison (Fig. 2B). We observed the expected distribution of the TKO3 library guides and calculated a mean of 339 reads per guide for a total of 24.0E6 reads (Fig. 2C). At an MOI of 1, we would expect ~ 37% of the cells to receive a single guide (~ 18% dual integration and ~ 8% > two integrations) yielding an average of 195 single integrations per guide. A parallel experiment of puromycin selection of the transduced iPSC-derived macrophage precursors with the TKOv3 CRISPR lentiviral library yielded an average of 64% transduced cells, further supporting the transduction efficiency of the pooled procedure. We identified 99.6–99.7% of the guides of TKOv3 library in our sequencing, depending on which replicate, and we detected an average of 89.1% reads mapping to only one guide of the TKOv3 library, with 10.8% reads not mapping to any guide and 0.09% of reads mapping to more than one guide (Fig. 2D), confirming that our protocol can be used to transduce a genome-wide library of guides in iPSC-derived macrophages (Reference sample deep sequencing data published on European Genome-Phenome Archive—https://ega-archive.org/). Our combination of experimental steps (polybrene, spinfection and Vpx) represents an optimised, efficient method to transduce individual or library CRISPR lentivectors into iPSC-macrophages to knock-out single genes as well as to perform genome-wide loss-of-function knockout screens.

## Discussion

**Figure 3:**
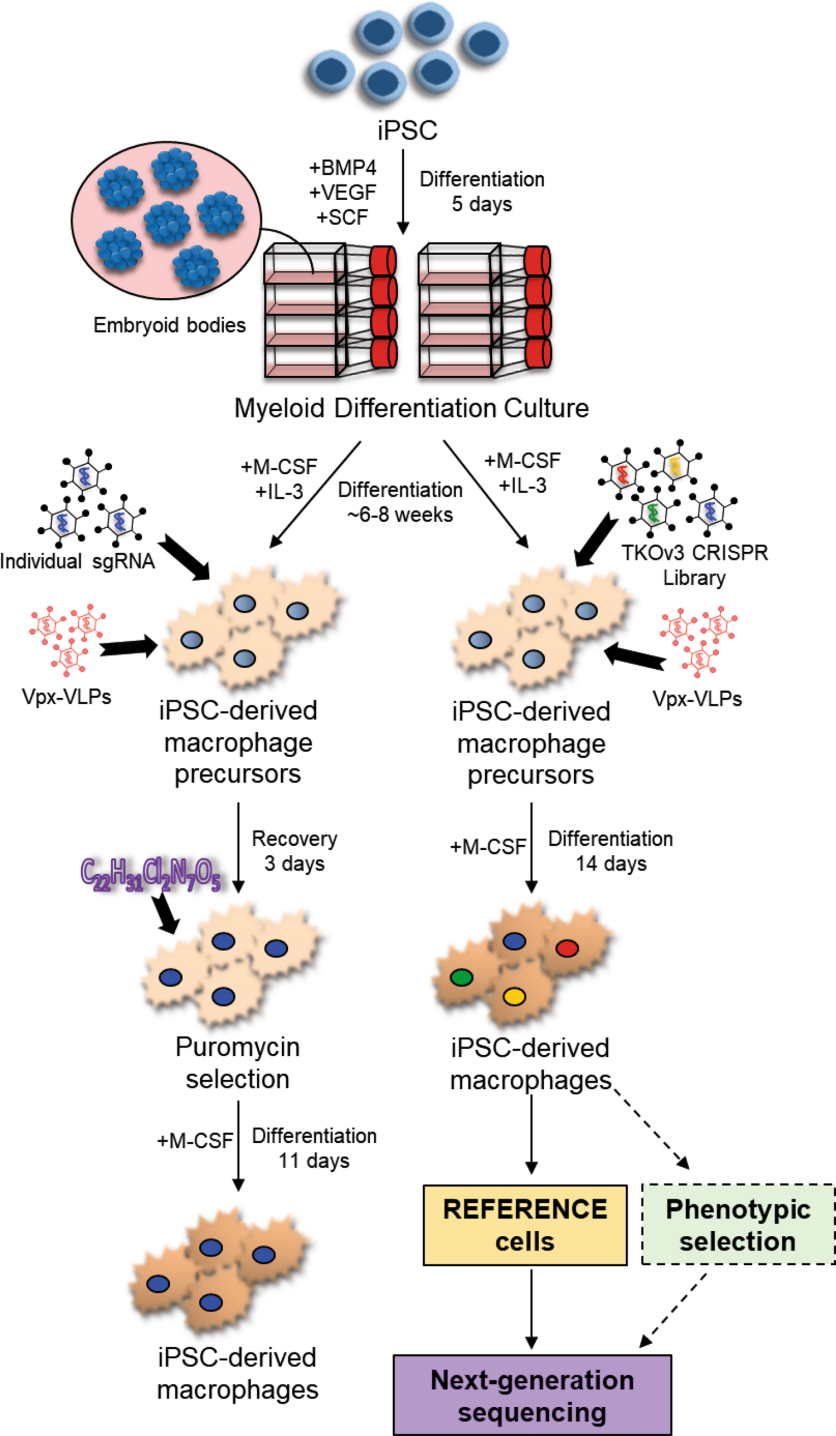
Lentiviral transduction for the delivery of CRISPR/CAS9 to macrophages derived from human iPSC. Embryoid bodies were generated from human iPSC in the presence of specific growth factors. The embryoid bodies were then differentiated in the presence of M-CSF and IL-3 until the emergence of precursor macrophages. Precursor macrophages were transduced with either lentivirus containing individual sgRNAs or the TKOv3 CRISPR lentiviral library in the presence of VPX-VLPs. For precursor macrophages transduced with individual guides, puromycin selection was started 3 days post-transduction and cells were allowed to differentiate to macrophages in M-CSF for another 11 days. For the precursor macrophages transduced with the TKOv3 library, cells were differentiated to macrophages in the presence of M-CSF for 14 days, after which the cells were collected for the Reference sample, genomic DNA extracted and analysed by next-generation sequencing. This protocol enables future application for genome-wide screens, for example based on phenotypic selection in the presence of the TKOv3 CRISPR lentiviral library (indicated by dashed lines).

Previous strategies have achieved efficient CRISPR/Cas9 knockout of single genes in iPSC-derived macrophages, however this procedure employed transient transfection of plasmid DNA to deliver Cas9 and the guide(s)^24^, thereby severely limiting their application to single gene knock-outs. Our method of lentiviral transduction (schematic of protocol shown in Fig. 3) has several advantages over transient transfection procedures; (i) enables stable expression of integrated vectors, (ii) can include sortable reporter markers, (iii) does not require additional, costly equipment, and (iv) is the delivery mechanism of choice for most genome-wide screens as the technique enables more precise control of the number of guides per cell. An alternative screening method was developed where iPSC lines^40^ were subjected to genome-wide mutagenesis by CRISPR/Cas9 library transduction and then differentiated to macrophages following an established macrophage differentiation protocol. Although efficient knock-out at the iPSC stage has been fairly well established for some time now^41^, these protocols are impractical when screening mature macrophages, a non-dividing cell model. Here we describe a method to transduce the cells at the macrophage stage and hence, we are able to express stable inserted guides and Cas9 while simultaneously avoiding the potential confounding problem of knocking-out genes important in the process of cell differentiation, a serious issue with macrophages^20^.

It is not possible to conduct clonal selection with our iPSC-derived macrophage model because like adult human macrophages, macrophages derived from iPSC do not proliferate, and as a consequence, the transduced edited cells are a heterogeneous population. Nevertheless, protein-based analysis confirmed that effective knock-outs of all proof-of-concept genes tested could be achieved; *HPRT1*, *PPIB* and *CDK4*.

Because inflammation has been implicated in many human pathologies, efficient CRISPR/Cas9-mediated gene-knockout methods in iPSC-derived macrophages are crucial for systematically exploring the molecular pathways that trigger the immune response. The improvement in the delivery of CRISPR/Cas9 technology that we describe in iPSC-derived macrophages will contribute to increasing the therapeutic potential of these cells. Future CRISPR screens can now be performed at genome-wide scale and in high-throughput with this valuable tool to identify key regulators of inflammation or other pathways regulated during the innate immune response.

## Methods

### Cloning of individual sgRNAs

Following Target Guide Sequence Cloning Protocol from Feng Zhang lab (LentiCRISPRv2: lentiviral CRISPR/Cas9 and single guide RNA^42^). See Table 1 for sgRNA sequences.

**Table 1:** sgRNA sequences.

sgRNA	Oligo sequence
HPRT1 guide#1	GTTATGGCGACCCGCAGCCC
HPRT1 guide#2	TCACCACGACGCCAGGGCTG
HPRT1 guide#3	GAAAGGGTGTTTATTCCTCA
HPRT1 guide#4	GATGTGATGAAGGAGATGGG
PPIB guide#1	CTTGCCGCCGCCCTCATCGC
PPIB guide#2	TGAAGTCCTTGATTACACGA
PPIB guide#3	TGTGGCCTTAGCTACAGGAG
PPIB guide#4	TTGCCGCCGCCCTCATCGCG
CDK4 guide#1	TTCGTGAGGTGGCTTTACTG
CDK4 guide#2	GTTTCGGCTGGCAAGCCTGG
CDK4 guide#3	CCAGATGGCACTTACACCCG
CDK4 guide#4	GTACCACCGACTGCACTGGG

### Cell culture lines

Human iPSC line SFC841-03-01 and SFC180-01-01 are previously published^23,27^. It was reprogrammed using Sendai reprogramming vectors (Cytotune, Life Technologies) from dermal fibroblasts from a healthy adult male donor, recruited through the Oxford Parkinson’s Disease Centre study: Participants were recruited to this study having given signed informed consent, which included derivation of hiPSC lines from skin biopsies (Ethics Committee: National Health Service, Health Research Authority, NRES Committee South Central, Berkshire, UK, who specifically approved this part of the study (REC 10/H0505/71).

### Generation of iPSC-derived macrophages

iPSC were cultured in a humidified environment at 37 °C with 5% CO_2_. iPSC were maintained in 2 mL of Essential 8 (E8) (ThermoFisher Scientific) media on Geltrex LDEV-Free Reduced Growth Factor Basement Membrane Matrix-coated plates (ThermoFisher Scientific) and passaged once achieving 70–80% confluence. In order to passage the cells, the cells were washed with PBS and incubated for 5 min at 37 °C in 0.5 mM EDTA in PBS. EDTA was removed and the cells were washed off the bottom of the well by adding 2 mL warm E8 media and pipetting gently up and down. The cells were distributed into new coated plates at an ~ 1:6 split and fed daily with 2 mL E8 media.

iPSC were differentiated into macrophages via Embryoid Body (EB) formation in AggreWell 800 (StemCell Technologies) plates. Cells were washed with PBS and incubated with 1 mL TrypLE Express (ThermoFisher Scientific) at 37 °C for 5 min. PBS was added to the TrypLE-treated cells and the cells were gently resuspended into a single cell suspension, collected into a centrifuge tube and diluted 1:10 with PBS. Cells were counted, spun down and resuspended in E8 media supplemented with 50 ng/mL BMP4 (ThermoFisher Scientific), 50 ng/mL VEGF (ThermoFisher Scientific), 20 ng/mL SCF (Miltenyi Biotec) and 10 ng/ml Rho kinase inhibitor Y27632 (Abcam) (this media is now referred to as EB media) at a concentration of 4.0E6 cells/mL. The AggreWell 800 plate was prepared by adding 0.5 mL of Anti-Adherence Rinsing Solution (StemCell Technologies) into each well and centrifuging the plate at 1000×*g* for 3 min in order to remove microscopic air bubbles. The Rinsing Solution was then removed and the wells were washed twice with PBS before adding 1 mL of EB media per well. This was followed by the addition of 1 mL of 4.0E6 iPSC into each well. The AggreWell 800 plate, with each well containing 4.0E6 iPSC in 2 mL of EB media, was centrifuged at 200×*g* for 3 min. The plate was checked to ensure that the cells were evenly distributed into the microwells and placed into the incubator. The AggreWell 800 plate was left for 4 days with daily media changes, where a p1000 pipette was used to gently aspirate 1 mL of culture media and 1 mL of fresh EB media was added slowly, in a drop-wise manner, to the side of the well to ensure that EBs were not washed out of the microwells. The EBs were harvested after 4 days by resuspending the contents of the wells using a 5 mL serological pipette to dislodge the EBs from the microwells and transferring to a 40 μm cell strainer (Corning) placed over a 50 mL centrifuge tube. The wells were washed out twice using PBS and the washes transferred to the same cell strainer. The strainer was then inverted and held at an angle over a new 50 mL centrifuge tube and the EBs were washed off the strainer into the tube. This was done by slowly passing through the strainer 10 mL of X-VIVO15 (Lonza) supplemented with 100 ng/mL M-CSF (ThermoFisher Scientific), 25 ng/mL IL-3 (ThermoFisher Scientific), 2 mM Glutamax (ThermoFisher Scientific), 100 U/mL penicillin/100 μg/mL streptomycin (ThermoFisher Scientific), and 0.055 mM β-mercaptoethanol (ThermoFisher Scientific) (this media is now referred to as myeloid differentiation media). The EBs from each well of the AggreWell 800 plate were transferred to a T175 flask containing 20 mL of myeloid differentiation media and left to differentiate, with 10–20 mL of fresh myeloid differentiation media added each week.

Macrophage precursors emerged into the supernatant from one month onwards and were harvested weekly. This was done by standing the flasks up, carefully collecting the supernatant from the flask without disturbing or collecting any floating EBs, and straining the supernatant through a 40 μm cell strainer. Fresh myeloid differentiation media was added back to the flask at a volume equal to the volume harvested in order to replenish the T175 flask. Harvested cells were plated onto tissue-culture treated plastic 12-well plates at 1.0E6 cells per well and differentiated for 7 days or more to mature macrophages in 1 mL per well of X-VIVO15 supplemented with 100 ng/mL M-CSF, 2 mM Glutamax, with media changes every 3–4 days.

### Vectors/libraries

lentiCRISPRv2 was a gift from Feng Zhang (Addgene plasmid # 52961; http://n2t.net/addgene:52961; RRID: Addgene_52961).

Toronto human knockout pooled library (TKOv3) was a gift from Jason Moffat (Addgene # 90294).

The plasmid pSIV3+ to produce lentivector VLP(+Vpx) was a gift from Jan Rehwinkel. pSIV3+ has been previously described^43^.

### Production of lentivectors, concentration and titration

The oligos of each guide were ordered, annealed and cloned into plentiCRISPRv2 following the authors’ protocol^1^ (See Table 1 for oligo sequences used). Clones were verified by Sanger sequencing. CRISPR clones and VPX-VLPs were co-transfected with packaging vectors psPAX2 (Addgene # 12260) and pCMV-VSV-G (Addgene # 8454) in equimolar ratios into HEK-293T cells to produce lentivirus. Production of lentivirus and TKOv3 library was performed following author’s protocol. Concentration of CRISPR and VPX lentivirus particles was done by ultracentrifugation at 29,000 rpm for 2 h at 4 °C, pellet was resuspended in PBS/BSA 1.5%, aliquoted and frozen at – 80 °C. The transduction efficiency of Vpx-VLPs was determined by using a range of cell numbers and different volumes of Vpx-VLPs. We identified that 2.5 µL per 1.0E6 cells was the minimum volume of VPX that eliminates SAMHD1 by western-blot. The CRISPR/Cas9 viruses were titered by spinfection of 1.0E6 iPSC-derived macrophage precursors per well in 24-well plates, with different dilutions of the virus in each well (and no virus control). Lentivirus were titered in presence of VPX-VLPs and PB. After puromycin selection, we determined the percentage of transduction by cell viability (after antibiotic resistance only infected cells survive puromycin) with a Resazurin test (Thermo Fisher Scientific). We calculated the number of transforming units (i.e. infectious LV particles) per microlitre, then we determined the viral volume that results in 50–60% of cells surviving in puromycin (MOI = 1).

### Lentiviral transduction for the delivery of CRISPR/Cas9 to macrophages derived from human iPSC

Precursor macrophages were transduced with either lentivirus containing individual sgRNAs or the TKOv3 CRISPR lentiviral library in the presence of polybrene (4 µg/mL) and VPX-VLPs in 12-well plates by spinfection at 800 g 2 h at 37 °C. For precursor macrophages transduced with individual guides, 1 µg/mL continuous puromycin selection was started 3 days post-transduction and cells were allowed to differentiate to macrophages in M-CSF for another 11 days. For the precursor macrophages transduced with the TKOv3 library, cells were differentiated to macrophages in the presence of M-CSF for 14 days, after which the cells were collected for the Reference samples. Genomic DNA extracted from these samples was then analysed by next-generation sequencing.

### Western analysis

Cells were washed with PBS and lysed in 1× RIPA Buffer (Thermo Fisher) with Halt Protease Inhibitor Cocktail (Thermo Fisher). Proteins were separated by size by SDS-PAGE with Bolt 4–12% Bis–Tris Plus Gel (Thermo Fisher) and blotted onto Whatman Protran nitrocellulose membrane (Sigma) using the Mini Trans-Blot Cell (Bio-Rad) according to the manufacturer’s instructions. Membranes were blocked in 4% skim milk in TBST. Antibodies to detect protein levels were incubated in 1% skim milk in PBST (Sigma) with primary antibody overnight. Membranes were washed with TBST and incubated in 1% skim milk in PBST with secondary antibodies for 1 h at RT. See Table 2 for immunoblotting antibodies.

**Table 2:** Antibodies (for flow cytometry and immunoblotting assays).

Antibody	Clone	Supplier	Cat. #	Dilution
**Flow cytometry**				
CD14-PE	HCD14	Biolegend	325605	1:20
IgG1κ-PE isotype control	MOPC21	Biolegend	400113	1:20
CD11b-Alexa Fluor 647	ICRF44	Biolegend	301319	1:20
CD80-Alexa Fluor 647	2D10	Biolegend	305215	1:20
IgG1κ-Alexa Fluor 647 Isotype control	MOPC21	Biolegend	400130	1:20
CD163-FITC	GHI/61	Biolegend	333617	1:20
IgG1κ-FITC isotype control	MOPC21	Biolegend	400109	1:20
TNF-Alexa Fluor 488	MAb11	BD Biosciences	557722	1:5
IgG1κ-Alexa Fluor 488 Isotype control	MOPC21	BD Biosciences	557721	1:5
**Immunoblotting**				
Cas9	7A9-3A3	Cell signaling	14697	1:1000
SAMHD1	Polyclonal	Abcam	ab67820	1:500
HPRT1	Polyclonal	Proteintech	15059-1-AP	1:500
PPIB	EPR12703	Abcam	ab178397	1:500
PPIB	Polyclonal	Abcam	ab16045	1:10
CDK4	Polyclonal	BioLegend	633301	1:500
CDK4	Polyclonal	GeneTex	GTX102993	1:10
GAPDH	G-9	scbt	sc-365062	1:1000
GAPDH	14C10	Cell signaling	2118	1:500
Alexa Fluor 750 goat anti-mouse	Polyclonal	Thermo Fisher	A-21037	1:5000
Alexa Fluor 680 goat anti-rabbit	Polyclonal	Thermo Fisher	A27042	1:5000
Anti-Rabbit detection module for wes	Polyclonal	Bio-techne	DM-001	ready-to-use

### PCR and TIDE

For TIDE, 0.5–2.0E6 cells were collected and DNA was purified using QIAamp DNA Micro Kit (QIAGEN). 300 ng total DNA from the sgRNA-targeted region was amplified using Q5 High-Fidelity DNA Polymerase (NEB). Amplicons were gel-purified using MinElute Kit (QIAGEN) and sent for sequencing at Eurofins. We used specific primers for each sgRNA-targeted region in WT and knockout samples.

For TKOv3 CRISPR Sequencing Library Preparation, we performed 2 PCRs to enrich guide-RNA regions in the genome and to amplify guide-RNA with Illumina TruSeq adapters following the author’s protocol. See Table 3 for a list of primers.

**Table 3:** Primers to amplify sgRNA-targeted region and for TIDE.

Primers	Sequence (5′–3′)
**PCR of sgRNA-targeted region**	
F-HPRT1 guides1 + 2	GCGGGGCCTGCTTCTC
R-HPRT1 guides1 + 2	CCTCCCCGCAGCACG
F-HPRT1 guide3	TGTAATGCTCTCATTGAAACAGC
R-HPRT1 guide3	TGCATAGCCAGTGCTTGAGA
F-HPRT1 guide4	GGGCAAAGGATGTGTTACGTG
R-HPRT1 guide4	AAGCAAGTATGGTTTGCAGAGA
F-PPIB guides1 + 4	GAGCCCGCGAGCAACC
R-PPIB guides1 + 4	CCGGGGATCCAACTAACTCC
F-PPIB guide2	TTTCCATTGAGCCAGCAGGT
R-PPIB guide2	ACGGTCACCTGGGAGAGATT
F-PPIB guide3	GAACTTAGGCTCCGCTCCTT
R-PPIB guide3	TCAGGGCCCCATTACTCTTA
F-CDK4_guides1 + 2 + 3	TGCAGGCTCATACCATCCTA
R-CDK4_guides1 + 2 + 3	GACCCCATGGGTTACCATGAA
F-CDK4_guide4	GGAAACTCTGAAGCCGACCA
R-CDK4_guide4	CTGTGGGTGGCTATTTGCAG
**TIDE**	
H1	CTGCTTCTCCTCAGCTTCAGGC
H3	GAGTCCCACTATACCACAACTGAC
R-HPRT1 guide4	AAGCAAGTATGGTTTGCAGAGA
P1_4	CACAGACAGAAGCCGAGGGA
P2	CGCTCACTTAGTAGCACACTGA
R-PPIB guide3	GAACTTAGGCTCCGCTCCTT
C1	CTGGCTGAAATTGGTGTCGGT
C2	GGTGGGGTGTGATGATCTGTAG
R-CDK4_guides1 + 2 + 3	GACCCCATGGGTTACCATGAA
C4	CTGAGTCCTCTTTCTGCTGAACC

### LPS stimulations

Cells were transduced with virus and differentiated for 14 days followed by stimulation with 100 ng/mL LPS (Sigma, L3024). After 30 min incubation with LPS, 1 μL of Brefeldin A (GolgiPlug, BD Biosciences) was added per 1 mL of culture media containing 1.0E6 cells, according to manufacturer’s instructions. After 6 h of LPS stimulation in the presence of Brefeldin A, the cells were washed with PBS and harvested by treating with 0.5 mM EDTA in PBS for 5–10 min at 37 °C.

### Flow cytometry

For analysis of cell surface markers, 1.0E6 cells were transduced with virus, differentiated for 14 days, EDTA-treated and incubated in PBS containing 1% BSA with either the primary antibody or an isotype-matched control (with the same fluorophore, from the same manufacturer), for 30 min on ice, in a final volume of 100 μL. For intracellular cytokine staining, cells were treated as above, except that cells were first fixed and permeabilised using a Cytofix/Cytoperm solution kit (BD Biosciences) before incubating in Perm/Wash Buffer (BD Biosciences) with primary antibody (or isotype-matched control) overnight at 4 °C, in a final volume of 25 μL. The cell state was analysed by staining with LIVE/DEAD Fixable Violet dye (ThermoFisher Scientific) according to manufacturer’s instructions. Fluorescence was measured using a Cyan II flow cytometer (Beckman Coulter) and the data was analysed using Summit v4.3. software (Beckman Coulter). All assays were run in triplicates. See Table 2 for a list of flow cytometry antibodies.

### Next-generation sequencing

The resulting PCR-amplified product was sequenced as 75 bp paired end reads using the Illumina HiSeq 4000. The adaptors were trimmed and the resulting sequences were mapped to the guide references using Bowtie2 (version 2.2.5) allowing up to one mismatch. Only reads which mapped to one guide were kept; which was on average 89.1% of the reads across the replicates. The guides were counted using Mageck (version 0.5.9.2).

### Statistics

Reference sample replicate correlation of TKOv3 library integration into iPSC macrophages of 70,948 guides targeting 18,053 protein-coding genes, 142 guides targeting LacZ, EGFP, and luciferase (controls) was conducted using Pearson’s Correlation Coefficient.

Validation experiments. To test the statistical significance of the results shown in Figs. 1, S1 and S2, we used a paired t-test. All experiments were performed in three or more replicates, and averages or one representative experiment is shown in all panels.

### Ethical statement

All experiments and methods were performed in accordance with relevant guidelines and regulations. All experimental protocols were approved by a named institutional/licencing committee. Specifically, Human iPSC line SFC841-03-01 and SFC180-01-01 are cell lines previously generated from participants with informed consent (Ethics Committee: National Health Service, Health Research Authority, NRES Committee South Central, Berkshire, UK), who specifically approved this part of the study (REC 10/H0505/71).

The iPSC lines used in this study are available through StemBANCC or the Oxford Parkinson’s Disease Centre subject appropriate consents and agreements. The investigator labs and commercial sources of all other reagents are described. Reference sample deep sequencing data published on European Genome-Phenome Archive—https://ega-archive.org/).

## Supplementary Information

Supplementary Information.
