# Supplementary material for: Genome-wide CRISPR/Cas9-knockout in human induced Pluripotent Stem Cell (iPSC)-derived macrophages
Source: Sci Rep. 2021 Feb 19;11:4245. doi: 10.1038/s41598-021-82137-z (PMC7895961; doi:10.1038/s41598-021-82137-z)
Supplement: Supplementary file 1 — Supplementary Information. [file 41598_2021_82137_MOESM1_ESM.pdf]

FIGURE S1

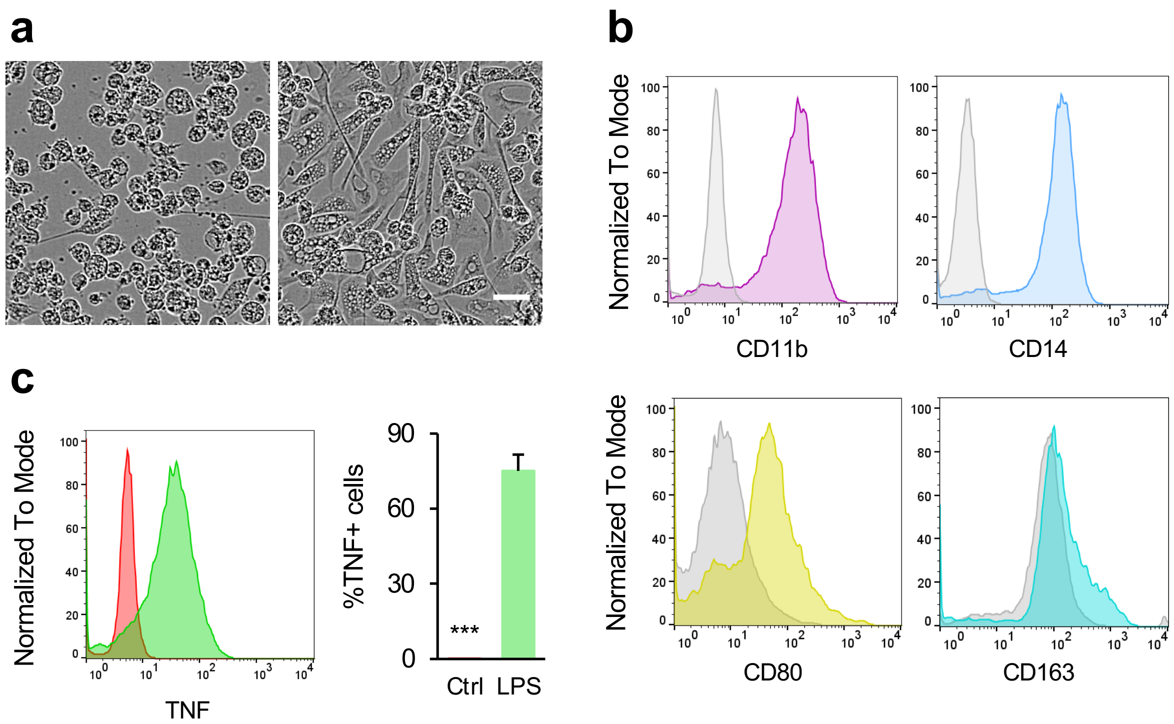

Supplementary Figure 1. Characterisation of iPSC-derived macrophages. A, Images of iPSC-derived macrophages precursors (left) and differentiated macrophages (right). B, Flow cytometry histograms showing expression of CD11b, CD14, CD80 and CD163 in differentiated iPSC-derived macrophages. Histograms show isotype control in grey. C, Flow cytometry histogram and Mean percent TNF$^+$ cells in control (red) and LPS-treated iPSC-derived macrophages (green) (±SEM; p-value=0.007; n=3). TNF histogram has been matched with isotype signal. Scale bar = 50µm

FIGURE S2

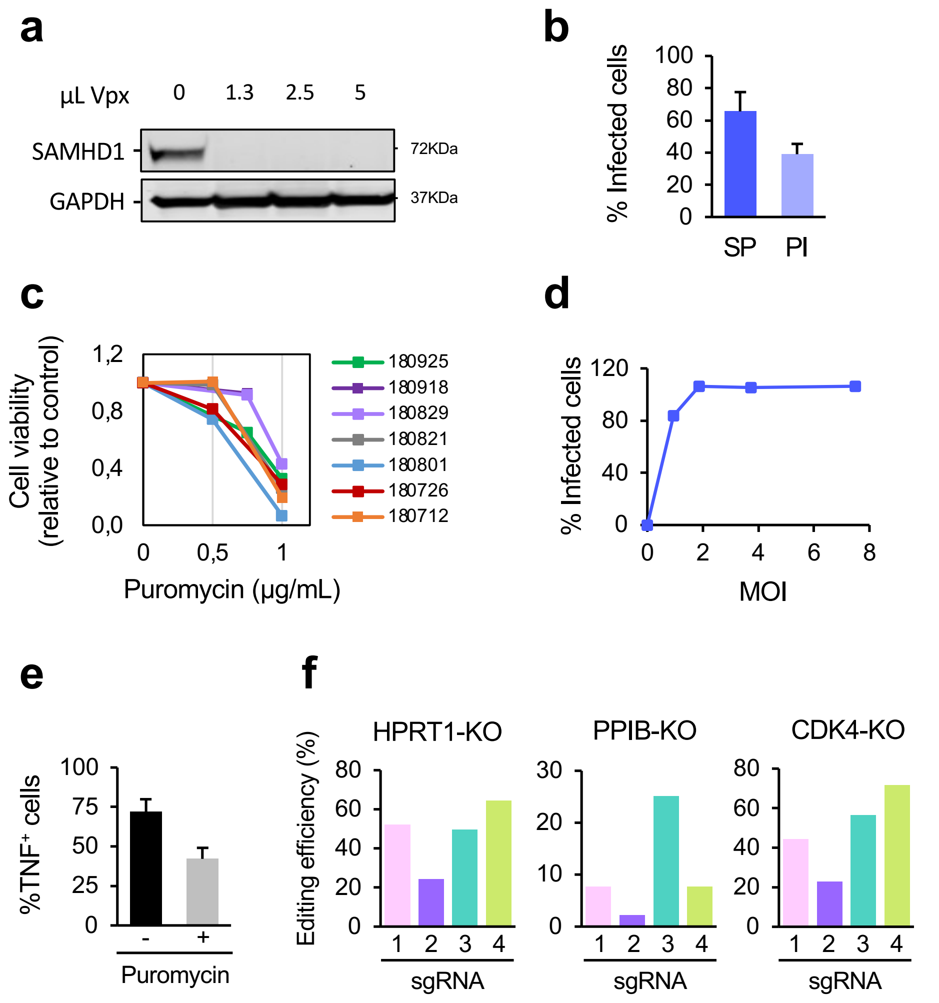

Supplementary Figure 2. CRISPR/Cas9 transduction and editing efficiencies in iPSC-derived macrophages. A, Western blot analysis for SAMHD1 expression in Vpx-transduced iPSC-derived macrophages using different volumes of Vpx-VLPs. Spinfection with PB. Display of cropped blots. GAPDH loading control. B, Percentage of transduced iPSC-derived macrophages after plate-infection (PI) or spinfection (SP) in presence of polybrene and VPX-VLPs (n=7; p-value=0.178). C, Cell viability relative to control (no puromycin) after treatment with different concentrations of puromycin, graph shows 7 samples. D, Transduction efficiency of TKOv3 lentiviral library in iPSC-macrophages. The percentage of infected cells show evidence of puro resistance in the transduced cells. E, Averaged percentage of TNF+ cells analysed by flow cytometry in iPSC-derived macrophages that were TKOv3 infected and LPS-treated (n=3; p-value=0.051). F, Editing efficiency measured by TIDE of each of the four guides used to knockout HPRT1, PPIB and CDK4 genes.

FIGURE S3

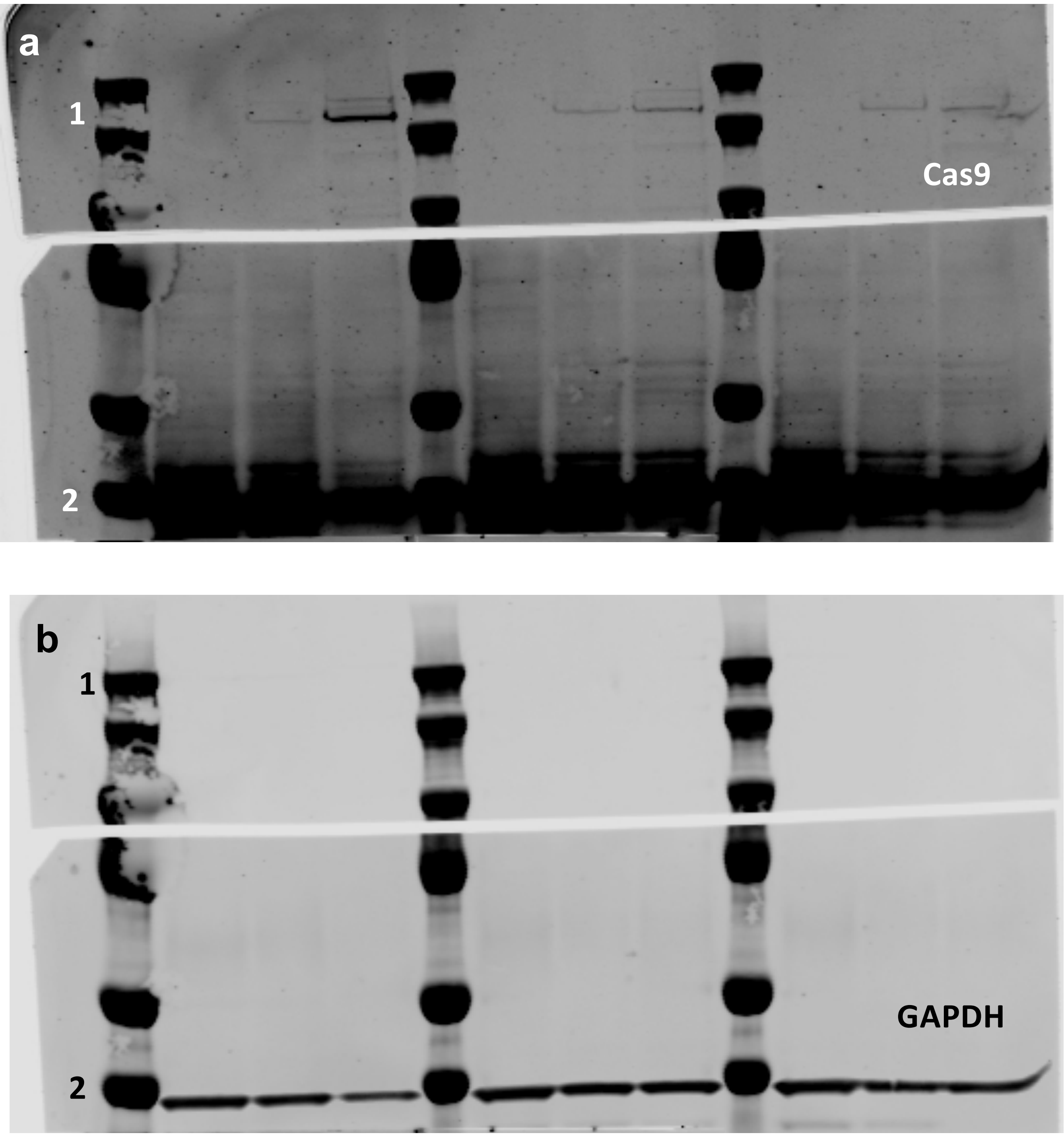

Supplementary Figure 3 . Western blot analysis of Cas9 protein in CRISPR/Cas9- transduced iPSC-derived macrophages. Full images of gel for Cas9 (a), and loading control GAPDH (b) levels, which were used to create main figure 1B of the main text with increasing exposure time, imaged on LI-COR. Gel is reconstructed here in full, it was cut to incubate different antibodies (numbers indicate the membrane cuts incubated separately with; 1, Cas9 antibody; and 2, GAPDH antibody).

FIGURE S4

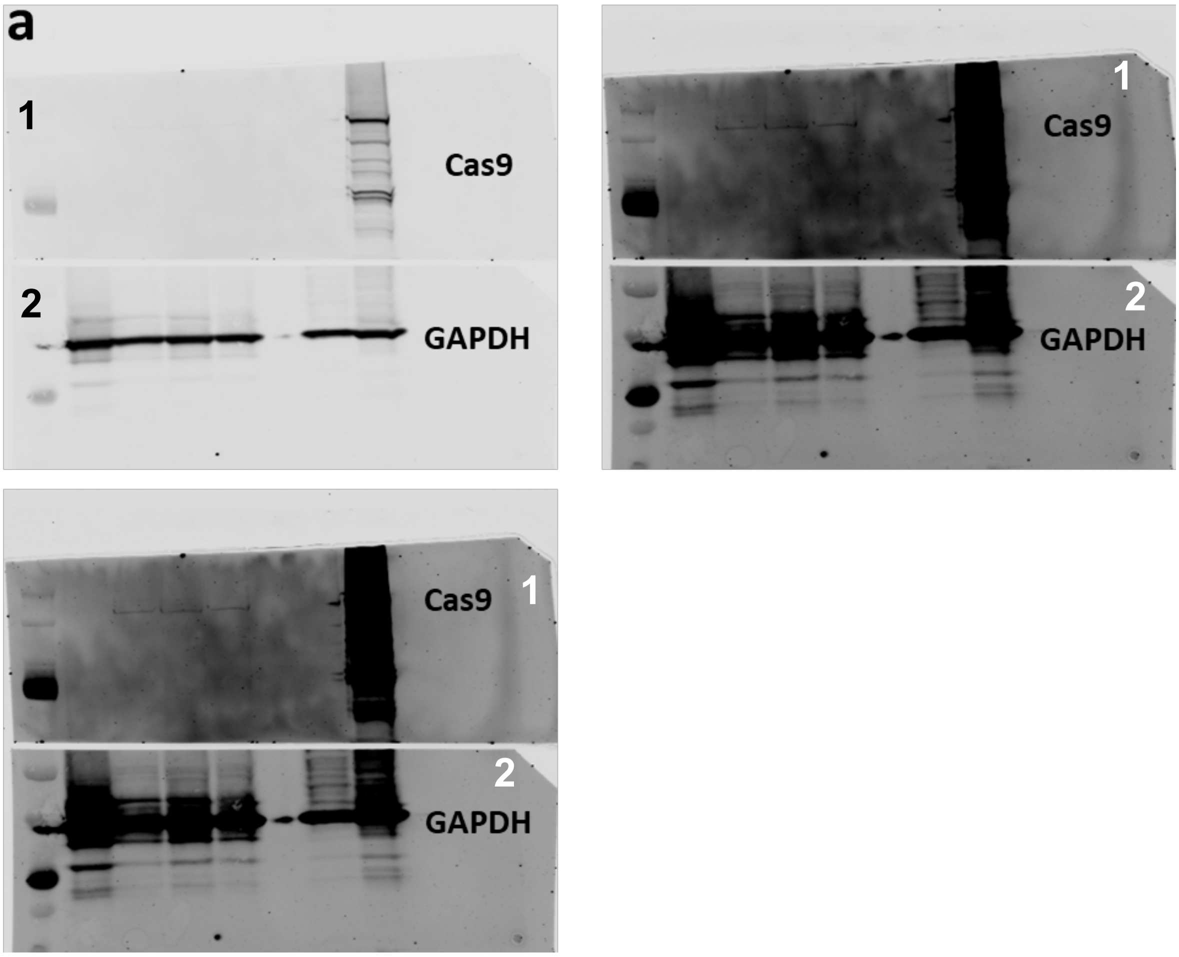

Supplementary Figure 4. Western blot analysis of Cas9 protein in TKOv3 library-transduced iPSC-derived macrophages. A, Western blot analysis of macrophages derived from iPSC. Full length gel for TKOv3 genome-wide library (Cas9 expression; 1) and loading control GAPDH (2), which were used to create main figure 2A of the main text, with increasing exposure time. Gel was cut to incubate different antibodies and is reconstructed here in full (numbers indicate the membrane cuts incubated separately with; 1, Cas9 antibody; and 2, GAPDH antibody).

FIGURE S5

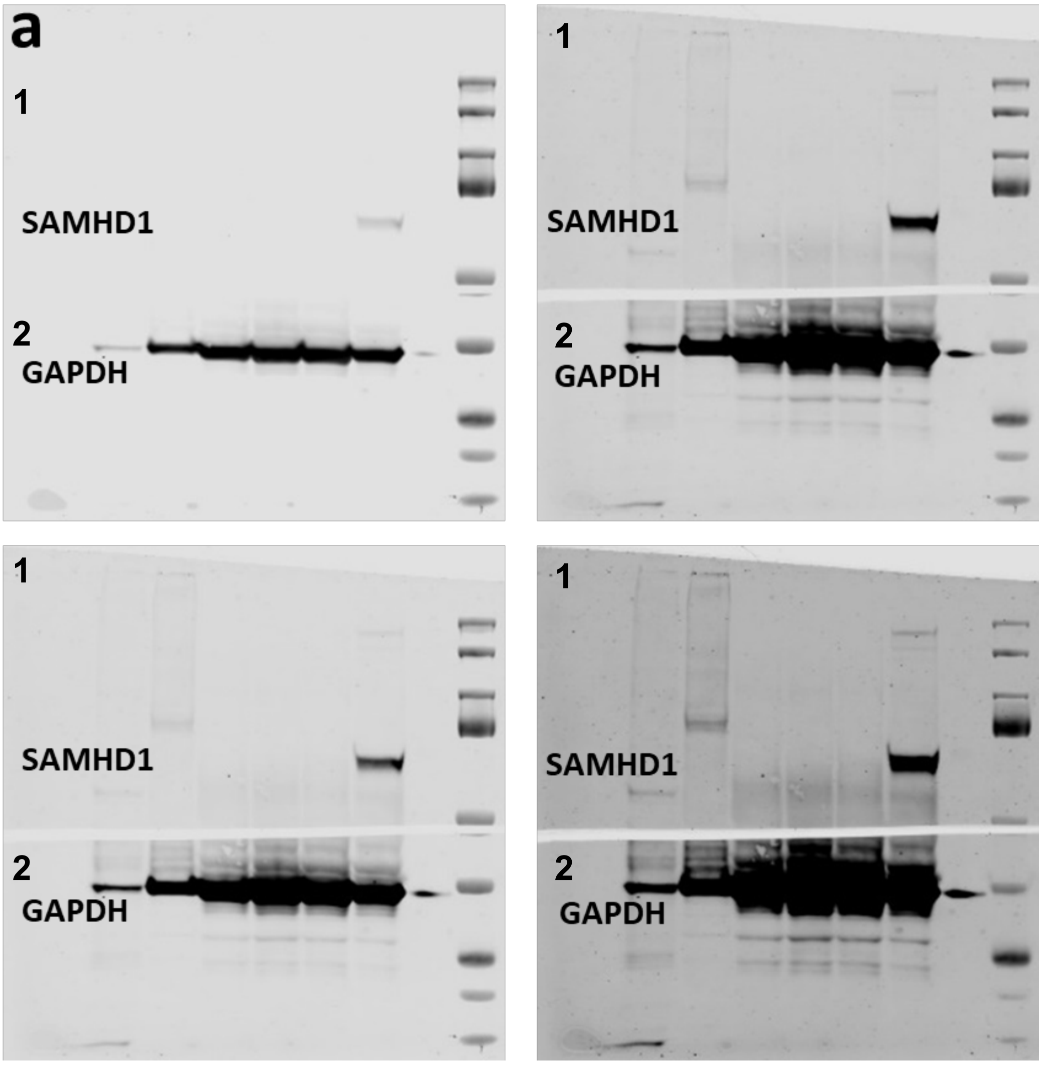

Figure Supplementary 5. Western blot analysis of SAMHD1 protein in VPX-VLP-transduced iPSC-derived macrophages. A, Western blot analysis of macrophages derived from iPSC. Full length gel for SAMHD1 (1) and loading control GAPDH (2), which were used to create Supplemental figure 2A, with increasing exposure time. In order to illustrate more clearly the VPX titter, we used a mirror image of this gel to create Fig. S2A. Gel was cut to incubate different antibodies and is reconstructed here in full (numbers indicate the membrane cuts incubated separately with; 1, SAMHD1 antibody; and 2, GAPDH antibody).

FIGURE S6

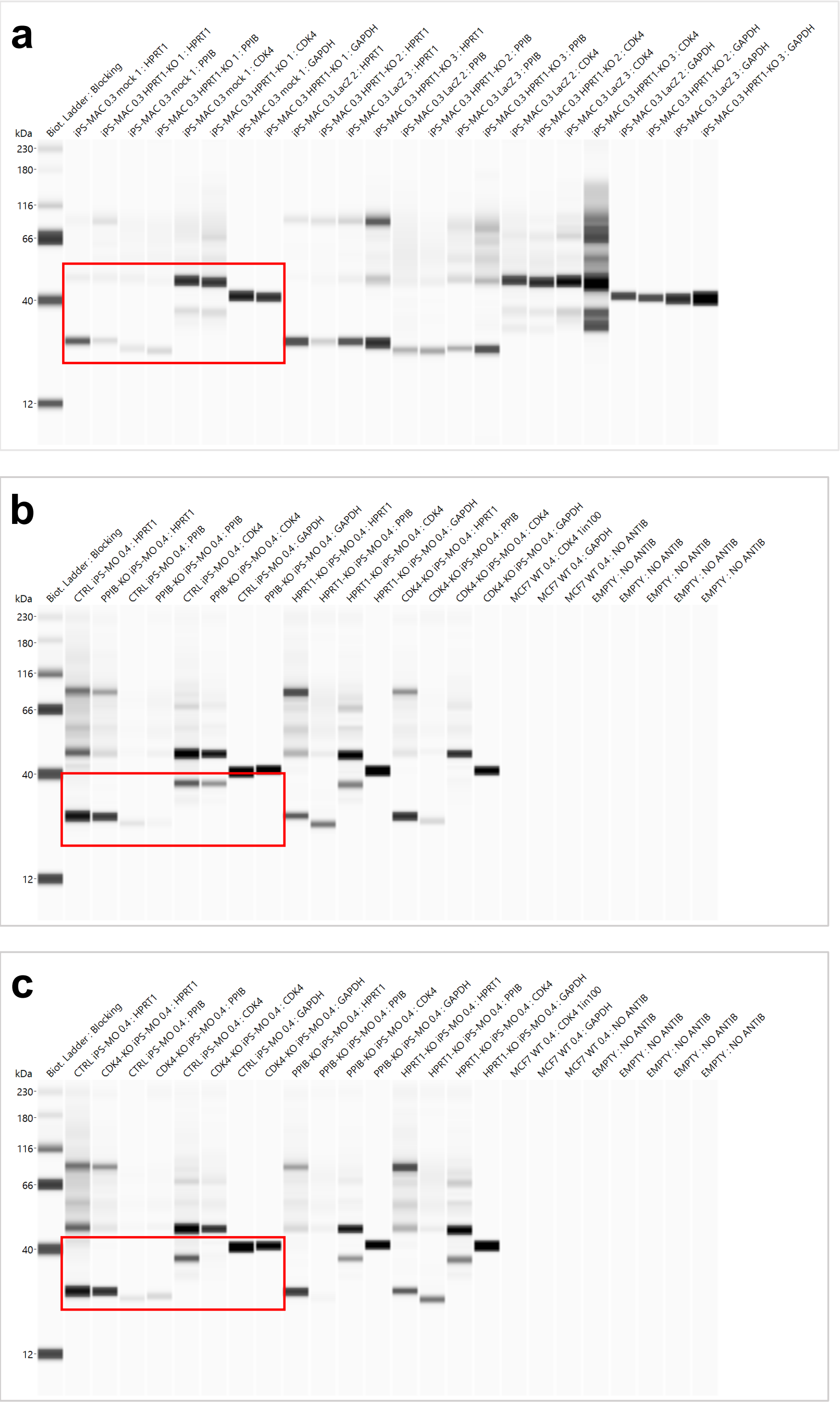

Supplementary Figure 6. Capillary Wes analysis of HPRT1, PPIB and CDK4 proteins in CRISPR/Cas9-transduced iPSC-derived macrophages. Full length capillary Wes gels of HPRT1-KO (A), PPIB-KO (B) and CDK4-KO (C) samples from figure 2D. The lanes showed in fig. 2D are highlighted in red squares. Loading control GAPDH. Capillary Wes images were obtained from Compass for SW 5.0.1. software. https://www.proteinsimple.com/compass
